# Evaluation of the durability and use of long‐lasting insecticidal nets in Nicaragua

**DOI:** 10.1186/s12936-021-03604-6

**Published:** 2021-02-19

**Authors:** Emperatriz Lugo Villalta, Aida Mercedes Soto Bravo, Lucrecia Vizcaino, Nicole Dzuris, Marco Delgado, Michael Green, Stephen C. Smith, Audrey Lenhart, Alexandre Macedo de Oliveira

**Affiliations:** 1 Entomología Médica, Ministerio de Salud, Managua, Nicaragua; 2 Medica Salubrista, Managua, Nicaragua; 3 Entomology Branch, Division of Parasitic Diseases and Malaria, Center for Global Health, Centers for Disease Control and Prevention, Atlanta, USA; 4 Malaria Branch, Division of Parasitic Diseases and Malaria, Center for Global Health, Centers for Disease Control and Prevention, Atlanta, USA

## Abstract

**Background:**

Vector control for malaria prevention relies most often on the use of insecticide-treated bed net (ITNs) and indoor residual spraying. Little is known about the longevity of long-lasting insecticidal nets (LLINs) in the Americas. The physical integrity and insecticide retention of LLINs over time were monitored after a bed net distribution campaign to assess community practices around LLIN care and use in Waspam, northeastern Nicaragua.

**Methods:**

At least 30 nets were collected at 6, 12, 24, and 36 months post distribution. Physical integrity was measured by counting holes and classifying nets into categories (good, damaged, and too torn) depending on a proportionate hole index (pHI). Insecticide bioefficacy was assessed using cone bioassays, and insecticide content measured using a cyanopyrethroid field test (CFT).

**Results:**

At 6 months, 87.3 % of LLINs were in good physical condition, while by 36 months this decreased to 20.6 %, with 38.2 % considered ‘too torn.’ The median pHI increased from 7 at the 6-month time point to 480.5 by 36 months. After 36 months of use, median mortality in cone bioassays was 2 % (range: 0–6 %) compared to 16 % (range: 2–70 %) at 6 months. There was a decrease in the level of deltamethrin detected on the surface of the LLINs with 100 % of tested LLINs tested at 12 months and 24 months crossing the threshold for being considered a failed net by CFT.

**Conclusions:**

This first comprehensive analysis of LLIN durability in Central America revealed rapid loss of chemical bioefficacy and progressive physical damage over a 36-month period. Use of these findings to guide future LLIN interventions in malaria elimination settings in Nicaragua, and potentially elsewhere in the Americas, could help optimize the successful implementation of vector control strategies.

## Background

In 2018, an estimated 764,980 cases of malaria occurred in the Americas, decreasing from 1.2 million in 2000 [1]. A total of 13,226 confirmed cases of malaria were reported in Nicaragua in 2019, of which 83 % were caused by *Plasmodium vivax*. The municipality of Waspam in the Autonomous Region of the North Atlantic is a municipality with a high risk of malaria transmission. In 2011, the year of this evaluation, more than 70 % of the total malaria reported in that region was reported in Waspam (pers. commun.). Malaria prevention and control in Nicaragua relies on proper malaria case management and vector control, mainly through the use of insecticide-treated bed nets (ITNs) and indoor residual spraying.

The Nicaraguan Ministry of Health has implemented integrated plans for malaria control with support from the Global Fund and other donors, including the distribution of long-lasting insecticidal nets (LLINs). LLINs offer protection from malaria by providing both a physical and a chemical barrier to mosquitoes seeking to feed on humans. A growing body of literature has described the durability and use of LLINs in multiple contexts in Africa. Previous research reported that after 38 months of use, polyester LLINs, such as PermaNet®, demonstrated a significant loss of both physical integrity and insecticide content [2]. This raised concerns about how long LLINs would remain functional under regular use and led to a series of studies regarding net distribution and replacement strategies, primarily in Africa and Asia [3–5]. Very little is known about the longevity of these tools in settings in the Americas.

As countries in the Americas increasingly focus on malaria elimination, it is of growing importance to understand the factors that can impact the optimal functioning of vector control interventions. The cultural practices and epidemiologic characteristics of malaria transmission in the Americas are distinct from Africa and may impact how LLINs are cared for and how long they are effective. As such, the objective of this study was to monitor the physical integrity and insecticide retention of LLINs over time, and to assess community practices around LLIN care and use in a malaria elimination setting in northeastern Nicaragua.

## Methods

### Study site

This study took place in Waspam municipality (14° 44′ 30.8″ N, 83°58′ 18.1″ W), located in the North Atlantic Autonomous Region (RAAN) of northeastern Nicaragua. Urban Waspam has an estimated 11,432 inhabitants and 1732 houses, and perennial malaria transmission.

### LLIN sampling strategy

A total of 1768 rectangular PermaNet 2.0® LLINs (160 × 180 × 150 cm, 100 denier, deltamethrin-treated) were distributed to children under 5 years of age and pregnant women in May 2010. This study followed the World Health Organization (WHO) guidelines for ITN evaluation with some modifications [6]. LLINs were collected at four different time points after distribution: 6, 12, 24, and 36 months. At least 30 nets were needed at each time point. To maximize the possibility of achieving the target sample sizes, a progressively greater number of houses were selected to have their LLINs collected at each time point: 55 houses for time points during the first year (at 6 months and 12 months), 65 houses at 24 months, and 75 houses at 36 months.

Houses were selected by simple random sampling among those that received at least one LLIN. There was no replacement for houses that could not be located, or if no LLIN was available for evaluation. In cases where more than one LLIN from the original distribution was present in a house, one was selected for collection by numbering nets and selecting a number from a cloth bag randomly. Replacement LLINs were provided for those collected during the study. At the time of LLIN collection, a survey collecting information on net care, use, and perceptions of the impact of the net in malaria prevention was administered.

### Physical durability of the LLINs

LLINs were transported to the Direccion de Entomologia Medica-Centro Nacional de Diagnostico y Referencia in Managua. Frames measuring 165 × 185 × 155 cm (corresponding to the size and shape of the LLINs) were constructed using commercial plastic pipes with a black cover added to facilitate the examination of the LLIN by providing a contrasting background [2]. LLINs were hung on the frame and the number, size, and position of holes on each panel were recorded.

All holes, including seam failures, were measured to estimate a proportionate Hole Index (pHI), based on WHO guidelines [7]. Holes were measured using the thumb, fist, head method. Hole sizes were estimated as follows: size 1, smaller than a thumb (0.5–2 cm); size 2, larger than a thumb but smaller than a fist (2–10 cm); size 3, larger than a fist but smaller than a head (10–25 cm); and size 4, larger than a head (> 25 cm).

The pHI was calculated by weighting each hole by size and summing for each net [8]. The weights correspond to the approximate hole areas of each hole size category (1.23 cm^2^, 28.28 cm^2^, 240.56 cm^2^, 706.95 cm^2^, respectively), divided by the smallest category of 1.23 cm^2^. These hole areas are based on the assumption that the hole sizes in each category are equal to the midpoints: pHI= (1 × no. of size-1 holes) + (23 × no. of size-2 holes) + (196 × no. of size-3 holes) + (576 × no. of size-4 holes). LLINs were then grouped into three categories based on pHI: good (≤ 64), damaged (65–642), and too torn (≥ 643) [9].

### Insecticide bioefficacy

**Fig. 1:**
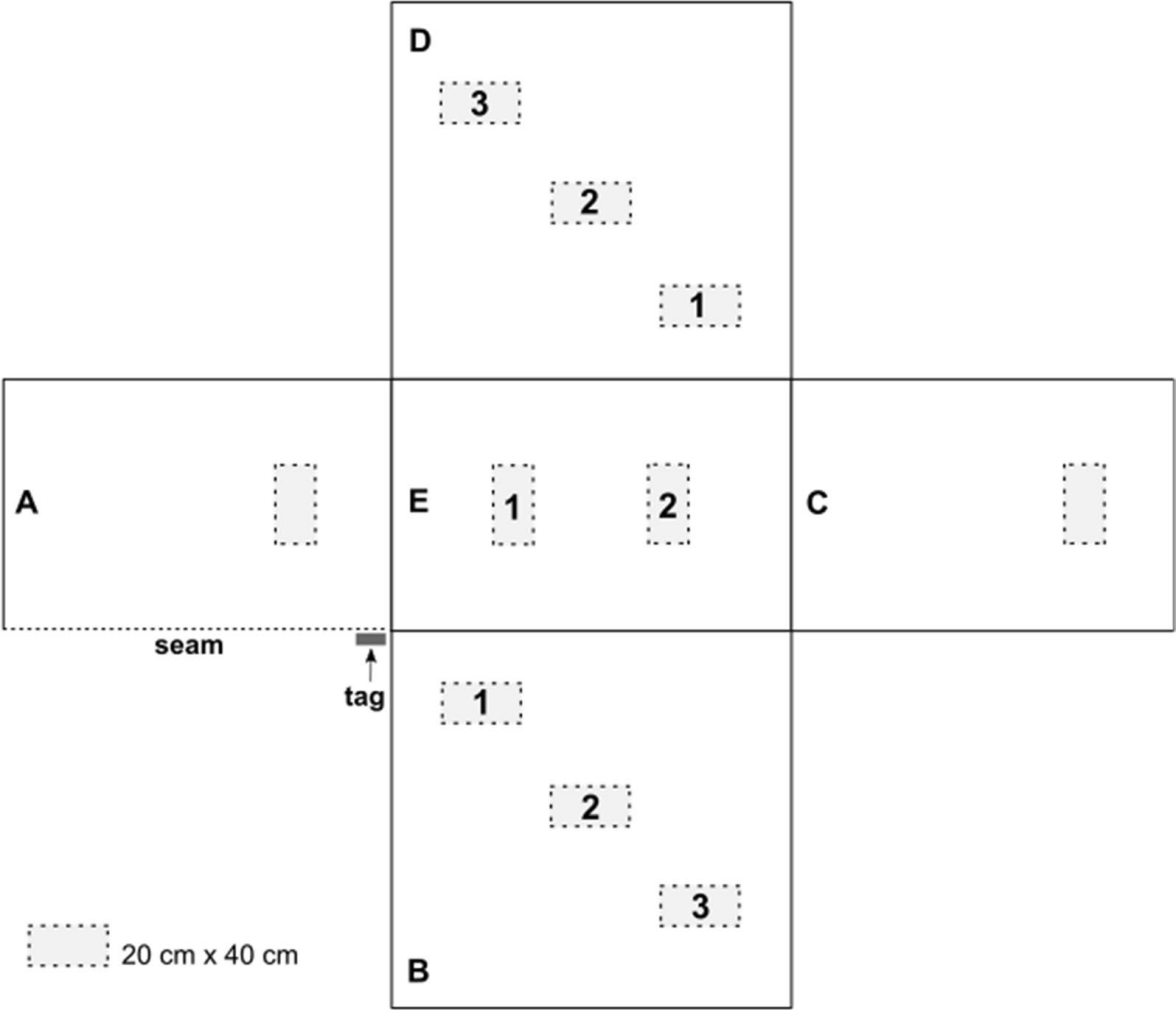
Diagram of the long-lasting insecticidal nets (LLINs) showing position of swatches collected for processing

After physical evaluation, a total of 10 swatches measuring 20 cm by 40 cm were cut from the different panels of the net (Fig. 1). Panels A and C corresponded to the ends of the net (head and foot panels), and swatches were cut 30 cm from the top center (panel A) or bottom center (panel C). On panels B and D (the lateral sides), three swatches were cut: the first one was 20 cm from both the side and from the top of the net, the second was 60 cm from the top at the center, and the third was 40 cm from the bottom and 20 cm from the side. Panel E was the roof of the net, and two swatches were cut from the center, 45 cm from the edge of panels A and C. Each swatch was then split in half, and one half was used for cone bioassays and the other half for the cyanopyrethroid field test (CFT) and high-performance liquid chromatography (HPLC) [10].

Cone bioassays were conducted using all 10 swatches from each LLIN following the WHO protocol [6]. Given the lack of an insecticide susceptible *Anopheles* colony, the Leningrado strain of *Aedes aegypti* was used in the cone bioassays. This was a local laboratory colony maintained at the Ministry of Health in Managua, which was tested using the CDC bottle bioassay and confirmed to be susceptible to deltamethrin. In summary, swatches were placed on a flat surface and a bioassay cone was securely attached to each piece. Five non-bloodfed female mosquitoes aged 2–5 days were introduced into each cone. Mosquitoes were exposed for three minutes and then transferred into holding containers. The knock down (KD) effect was recorded at 60 min, and mortality was recorded 24 h after exposure. The percent mortality was calculated from the proportion of dead mosquitoes relative to the total number of mosquitoes exposed. A control using an untreated bed net was run each day.

### High‐performance liquid chromatography (HPLC)

Twenty-six swatches, all from panel B3 collected at 6 months, were sent to CDC to perform HPLC. Prior to HPLC analysis, deltamethrin was extracted from five pieces measuring 5 cm × 5 cm taken from each swatch. The pieces were weighed together, submerged in 50 ml of solvent (20 % 1.4-dioxane, 80 % isooctane), and then placed in an ultrasound bath for 15 min, after which the flasks were shaken for 30 min in a 25°C bath (frequency of 155 cycles/min).

Analysis was conducted using an Agilent 1200 HPLC machine (Santa Clara, CA, USA) equipped with a 250 × 4 (i.d.) mm, LiChrosorb Si60 5µm, with iso-octane/1,4-dioxane (+ 0.15 % water), 94/6 (v/v) mobile phase at 1.5 ml/min flow rate. The total deltamethrin content was estimated using a UV detector at 230 nm [11].

### Surface insecticide retention

CFT was performed as previously described [12]. In summary, a magnetic sampling device (MSD) was prepared to obtain insecticide samples from the surfaces of swatches collected at each time point (6, 12, and 24 months). Pieces of filter paper were attached to the MSD, which exerted a constant force as the filter papers were systematically rubbed across both sides of the net surface within the confines of a wooden frame. The filter papers were placed into 24-well plates and the colorimetric reagent was added. The intensity of the purple colour formed from the reaction of deltamethrin with the colorimetric reagent was recorded with a digital camera and subsequently quantified using image analysis software. Each plate had a series of standard curve samples from which the amount of deltamethrin adhering to the papers per area of net sampled (mg/m^2^) was calculated. An average response factor was determined from the standard curve samples from each batch and used to determine deltamethrin surface levels. Batch assay precision (% relative standard deviation, %RSD) was determined by recalculating the standard curve samples from each plate using the associated batch response factor. The average %RSDs are 12, 7, and 6 % for 0.04, 0.10, and 0.20 mg/m^2^, respectively (n = 4 batches).

### Data analysis

Results from household (HH) surveys and laboratory data were double entered into an EpiInfo 2002 (Version 3.5.4, CDC, Atlanta, USA) database. Data cleaning and analyses were performed using RStudio (version 1.2.5033). Descriptive data are displayed as percentages for categorical variables and means or medians for continuous variables. Figures were generated using the ggplot2 package in R (version 3.1.0) and refined in Inkscape (version 0.92.3) [13, 14].

ANOVA and chi-square tests were conducted to determine significant differences in means and frequencies, respectively, between groups. Post-hoc Tukey honest significant difference (HSD) tests were used to determine where differences lie after statistically significant ANOVA tests. For a multivariate model of variables contributing to the physical degradation of LLINs, LASSO (least absolute shrinkage and selection operator) was performed using the glmnet package in R [15]. Cross validation was used to choose the shrinkage parameter. Variables of interest included length of time of use (estimated by collection time point), washing (method, frequency, water source, and drying location), wall material of the house, education level, number of people sleeping in the home, LLIN repairs, whether there were children under five in the home, and whether there were pregnant women in the home that slept under a net the night before. A 5 % level of significance was used for all statistical tests.

### Ethical considerations

All data collected as part of this evaluation were only accessible to those directly involved with the project. Participants’ verbal consent was obtained before the HH survey and LLIN collection. The protocol was approved as a program evaluation by the Office of the Associate Director for Science in the Center for Global Health at the US Centers for Disease Control and Prevention (CDC). The Nicaragua Ministry of Health also evaluated the project and deemed it programmatic evaluation.

## Results

### Household surveys and LLIN collection

From the 250 HHs originally selected, surveys were conducted in 165 (66.0 %): 55 at the first time point, 42 at the second, and 34 at both the third and fourth because not always a HH member could be found at the time of the visit. Of the 165 HH surveys conducted, 163 (98.8 %) HHs had at least one eligible LLIN available for collection. The median number of total sleeping spaces in all visited HHs the night before the survey was 4 (range: 0–14) and mostly consisted of beds (median = 3; range: 0–8) (Table 1). In 26 (15.8 %) HHs, a hammock had also been used for sleeping on the night preceding the survey. A total of 1015 people slept in the HHs the night before the survey (median = 5.0 per HH; range: 0–21). Of those, 429 (42.3 %) people slept under a campaign LLIN, 148 (34.5 %) were male, and the median age was 9 years (range: 1–80). Many HHs (78.2 %) reported having children under 5. Few HHs (6.7 %) reported at least one pregnant woman having slept under a campaign LLIN the night before.

**Table 1:** Details on sleeping spaces and campaign LLIN use by collection time point, Waspam, Nicaragua (n = 165 households)

Indicator	Collection time point				Total
	**6 months**	**12 months**	**24 months**	**36 months**	
Sleeping spaces, median (range)					
Beds	3 (0–8)	3 (1–6)	3 (0–8)	3 (1–7)	3 (0–8)
Hammocks	0 (0–2)	0 (0–2)	0 (0–1)	0 (0–1)	0 (0–2)
Any sleeping space	3 (0–8)	3 (1–8)	4 (2–14)	4 (1–12)	4 (0–14)
People sleeping in HH the night before, median (range)					
< 5 years old	1 (0–3)	1 (0–3)	1 (0–3)	1 (0–4)	1 (0–4)
5–15 years old	2 (0–10)	1 (0–4)	2 (0–9)	2 (0–5)	2 (0–10)
> 15 years old	3 (1–12)	1 (0–6)	3.5 (2–8)	3 (1–9)	3 (0–12)
All ages	8 (1–21)	2 (0–8)	6 (4–14)	6 (3–16)	5 (0–21)
People sleeping under a campaign LLIN the night before, % (n/N)	44.6 (189/424)	86.7 (91/105)	39.2 (93/237)	22.5 (56/249)	42.3 (429/1015)
Age, median (range)	11 (1–80)	6 (1–56)	13 (1–57)	24 (2–68)	9 (1–80)
Male, n (%)	62 (32.8)	34 (37.4)	37 (39.8)	15 (26.8)	148 (34.5)
HHs with ≥ 1 child under 5, % (n/N)	85.5 (47/55)	64.3 (27/42)	82.4 (28/34)	79.4 (27/34)	78.2 (129/165)
HHs with ≥ 1 child under 5 sleeping under a campaign LLIN the night before, % (n/N)	74.5 (35/47)	92.6 (25/27)	75.0 (21/28)	29.6 (8/27)	69.0 (89/129)
HHs with ≥ 1 pregnant woman sleeping under a campaign LLIN the night before, % (n/N)	0 (0/55)	0 (0/42)	5.9 (2/34)	26.5 (9/34)	6.7 (11/165)

The median number of bed nets per HH prior to the 2010 LLIN distribution was 3.0 (range: 0–12), while post-campaign this number increased to 5.0 (range: 0–12). Interviewees commonly reported that they learned about the LLIN distribution campaign via community leaders (40.0 %), followed by health promoters (36.4 %) or the radio (18.8 %). Socio-economic indicators associated with surveyed HHs are listed in Table 2.

**Table 2:** Socio-economic assets of LLIN owners by collection time point, Waspam, Nicaragua (n = 165 households)

Indicator	Collection time point				Total
	**6 months**	**12 months**	**24 months**	**36 months**	
Household asset present, n (%)					
Electricity	47 (85.5)	35 (83.3)	30 (88.2)	31 (91.2)	143 (86.7)
Radio	40 (72.7)	31 (73.8)	25 (73.5)	26 (76.5)	122 (73.9)
Television	39 (70.9)	33 (78.6)	28 (82.4)	20 (58.8)	120 (72.7)
Refrigerator	27 (49.1)	25 (59.5)	20 (58.8)	13 (38.2)	85 (51.5)
Bicycle	28 (50.9)	20 (47.6)	16 (47.1)	9 (26.5)	73 (44.2)
Motorcycle	6 (10.9)	13 (31.0)	6 (17.6)	1 (2.9)	26 (15.8)
Employment type, n (%)					
Self-employed	17 (30.9)	15 (35.7)	18 (52.9)	22 (64.7)	72 (43.6)
State institution	16 (29.1)	15 (35.7)	10 (29.4)	12 (35.3)	53 (32.1)
Family farm	9 (16.4)	0 (0.0)	1 (2.9)	9 (26.5)	19 (11.5)
Private institution	7 (12.7)	6 (14.3)	5 (14.7)	1 (2.9)	19 (11.5)
Nongovernmental organization	5 (9.1)	0 (0.0)	0 (0.0)	2 (5.9)	7 (4.2)
Housewife	0 (0.0)	0 (0.0)	0 (0.0)	3 (8.8)	3 (1.8)
Other	1 (1.8)	2 (4.8)	0 (0.0)	0 (0.0)	3 (1.8)
Unemployed	2 (3.6)	3 (7.1)	0 (0.0)	0 (0.0)	5 (3.0)
Principal water supply, n (%)					
Drilled well	30 (54.5)	26 (61.9)	21 (61.8)	22 (64.7)	99 (60.0)
Water pipes	19 (34.5)	9 (21.4)	11 (32.4)	8 (23.5)	47 (28.5)
River water	3 (5.5)	4 (9.5)	0 (0.0)	1 (2.9)	8 (4.8)
Public water stand or faucet	3 (5.5)	3 (7.1)	2 (5.9)	1 (2.9)	9 (5.5)
Rainwater in barrels	0 (0.0)	0 (0.0)	0 (0.0)	2 (5.9)	2 (1.2)
Principal hygiene service, n (%)					
Covered latrine	53 (96.4)	41 (97.6)	33 (97.1)	32 (94.1)	159 (96.4)
No facility, bush or field	1 (1.8)	0 (0.0)	0 (0.0)	2 (5.9)	3 (1.8)
Toilet	0 (0.0)	1 (2.4)	1 (2.9)	0 (0.0)	2 (1.2)
Uncovered latrine	1 (1.8)	0 (0.0)	0 (0.0)	0 (0.0)	1 (0.6)
Type of floor in home, n (%)					
Wood	48 (87.3)	33 (78.6)	28 (82.4)	27 (79.4)	136 (82.4)
Tile	3 (5.5)	4 (9.5)	6 (17.6)	3 (8.8)	16 (9.7)
Brick	4 (7.3)	5 (11.9)	0 (0.0)	1 (2.9)	10 (6.1)
Other	0 (0.0)	0 (0.0)	0 (0.0)	3 (8.8)	3 (1.8)
Type of walls in home, n (%)					
Wood	50 (90.9)	31 (73.8)	28 (82.4)	24 (70.6)	133 (80.6)
Blocks	5 (9.1)	7 (16.7)	3 (8.8)	1 (2.9)	16 (9.7)
Wood and blocks	0 (0.0)	4 (9.5)	2 (5.9)	5 (14.7)	11 (6.7)
Bamboo	0 (0.0)	0 (0.0)	1 (2.9)	4 (11.8)	5 (3.0)
Type of roof, n (%)					
Aluminum (zinc sheets)	55 (100.0)	40 (95.2)	33 (97.1)	34 (100.0)	162 (98.2)
Palm	0 (0.0)	2 (4.8)	1 (2.9)	0 (0.0)	3 (1.8)

All HHs surveyed (n = 165) reported having used the campaign LLINs at least once, and 97.6 % reported using them every night. Year-round use was reported by 93.9 % of HHs, while 5.5 % reported only using the net during the rainy season. Nearly all HHs surveyed (99.4 %) felt that bed nets were effective, even if their nets were torn or damaged. Most respondents (80.6 %) felt bed nets were important for malaria prevention, 44.8 % for protection from mosquitoes, and 1.8 % for diarrhea prevention. These frequencies did not differ by pHI category (p > 0.05). Stratifying data by collection time point, the proportion of respondents who perceived malaria prevention as a benefit of nets was highest at 12 months (88.1 %) and 24 months (94.1 %), and lowest at 36 months (58.8 %) (p = 0.001). The proportion of those who perceived protection from mosquitoes as a benefit was highest at six months (65.5 %) and 36 months (67.6 %) and lowest at 12 months (9.5 %) (p < 0.0001).

### Washing and care of LLINs

Of the 165 HHs surveyed, 162 (98.2 %) reported that their campaign LLINs had been washed at least once (Table 3). Greater than once per month was the most common washing frequency (45.1 %), and the most common washing methods involved using a cement wash basin and a wooden washboard/pan. Respondents reported that 159 (98.1 %) nets were washed using soap. Among those using soap, 77.4 % used a soap bar or ball, 13.2 % used powdered detergent, 8.8 % used a combination of powdered detergent and a soap bar/ball, and 0.6 % used liquid soap. After washing, 71.0 % of nets were dried outside in direct sunlight, many on wire fences (20.4 %).

**Table 3:** LLIN care practices by collection time point, Waspam, Nicaragua (n = 165 households)

Indicator	Collection time point*				Total
	**6 months**	**12 months**	**24 months**	**36 months**	
Washing Frequency, n (%)					
Ever washed	52/55 (94.5)	42 (100)	34 (100)	34 (100)	162/165 (98.2)
> Once per month	28/52 (53.8)	21 (50.0)	14 (41.2)	10 (29.4)	73/162 (45.1)
Once per month	17/52 (32.7)	17 (40.5)	7 (20.6)	16 (47.1)	57/162 (35.2)
Once per 6 months	4/52 (7.7)	1 (2.4)	10 (29.4)	7 (20.6)	22/162 (13.6)
Once per year	1/52 (1.9)	1 (2.4)	3 (8.8)	1 (2.9)	6/162 (3.7)
Unsure	2/52 (3.8)	2 (4.8)	0 (0.0)	0 (0.0)	4/162 (2.5)
Washing manner, n (%)					
Stone sink	34/52 (65.4)	26 (61.9)	19 (55.9)	1 (2.9)	80/162 (49.4)
Wooden washboard	6/52 (11.5)	13 (31.0)	14 (41.2)	25 (73.5)	58/162 (35.8)
By hand	12/52 (23.1)	1 (2.4)	1 (2.9)	8 (23.5)	22/162 (13.6)
River rock	0/52 (0.0)	1 (2.4)	0 (0.0)	0 (0.0)	1/162 (0.6)
Unsure	0/52 (0.0)	1 (2.4)	0 (0.0)	0 (0.0)	1/162 (0.6)
Soaked, n (%)					
Yes	42/52 (80.8)	37 (88.1)	29 (85.3)	27 (79.4)	135/162 (83.3)
No	9/52 (17.3)	5 (11.9)	5 (14.7)	6 (17.6)	25/162 (15.4)
Unsure	1/52 (1.9)	0 (0.0)	0 (0.0)	1 (2.9)	2/162 (1.2)
Soap used, n (%)					
Yes	49/52 (94.2)	42 (100.0)	34 (100.0)	34 (100.0)	159/162 (98.1)
Soap type, n (%)					
Bar or ball	40/49 (81.6)	29 (69.0)	29 (85.3)	25 (73.5)	123/159 (77.4)
Powdered detergent	8/49 (16.3)	3 (7.1)	1 (2.9)	9 (26.5)	21/159 (13.2)
Bar or ball, and powdered detergent	0/49 (0.0)	10 (23.8)	4 (11.8)	0 (0.0)	14/159 (8.8)
Liquid	1/49 (2.0)	0 (0.0)	0 (0.0)	0 (0.0)	1/159 (0.6)
Drying, n (%)					
Outside in the sun	43/52 (82.7)	28 (66.7)	24 (70.6)	20 (58.8)	115/162 (71.0)
Outside in the shade	9/52 (17.3)	13 (31.0)	7 (20.6)	9 (26.5)	38/162 (23.5)
In the home	0/52 (0.0)	1 (2.4)	3 (8.8)	5 (14.7)	9/162 (5.6)
Dried on wire, n (%)	4/52 (7.7)	7 (16.7)	15 (44.1)	7 (20.6)	33/162 (20.4)
Water source for washing LLINs, n (%)					
Drilled well	35/52 (67.3)	20 (47.6)	21 (61.8)	23 (67.6)	99/162 (61.1)
Drinking water pipes	12/52 (23.1)	10 (23.8)	7 (20.6)	4 (11.8)	33/162 (20.4)
River water	5/52 (9.6)	12 (28.6)	6 (17.6)	7 (20.6)	30/162 (18.5)

* At 6 months, only 52 out of 55 LLINs collected had been washed, while all LLINs collected at 12 (42 LLINs), 24 (34 LLINs), and 36 (34 LLINs) months had been washed when recollected

### Physical condition

Over the four collection time periods, a total of 2,816 holes were counted: 2,119 (75.2 %) size 1, 511 (18.1 %) size 2, and 186 (6.6 %) size 3 (Table 4). No LLINs collected as part of this study had size 4 holes (> 25 cm). The large lateral side panels had the most holes, with 27.0 % and 27.1 % of holes counted on panels B and D, respectively (Table 4). The head (A) and foot (C) panels had 16.4 % and 17.8 % of holes, respectively, and the roof had the least, with 11.8 % of holes. The highest concentration of holes was near the bottom of the net. In this study, 62.9 % of side panels with holes had most of the damage in the bottom third of the net; 16.9 % of panels had most of the damage in the middle third, 13.2 % in the top third, and 7.0 % had damage equally distributed across the panel.

**Table 4:** Hole count and location of most damage on panel of LLINs collected at all time points, Waspam, Nicaragua (n = 163 nets)

Indicator	Panel					Total
	**A**	**B**	**C**	**D**	E*	
Holes, n (%)						
Size 1	331 (71.8)	577 (75.9)	384 (76.8)	589 (77.3)	238 (71.5)	2,119 (75.2)
Size 2	100 (21.7)	136 (17.9)	81 (16.2)	115 (15.1)	79 (23.7)	511 (18.1)
Size 3	30 (6.5)	47 (6.2)	35 (7.0)	58 (7.6)	16 (4.8)	186 (6.6)
Total	461 (100.0)	760 (100.0)	500 (100.0)	762 (100.0)	333 (100.0)	2,816 (100.0)
Location of most damage on panel, n (%)						
Top third	10 (10.9)	14 (12.7)	14 (17.1)	13 (12.9)	21 (28.4)	72 (15.7)
Middle third	14 (15.2)	15 (13.6)	17 (20.7)	19 (18.8)	22 (29.7)	87 (19.0)
Bottom third	63 (68.5)	70 (63.6)	46 (56.1)	63 (62.4)	24 (32.4)	266 (58.0)
Equal distribution	5 (5.4)	11 (10.0)	5 (6.1)	6 (5.9)	7 (9.5)	34 (7.4)
Total	92 (100.0)	110 (100.0)	82 (100.0)	101 (100.0)	74 (100.0)	459 (100.0)

*For the roof (E) top third = along panel A, bottom third = along panel C

Hole size categories: size 1 (0.5–2 cm), size 2 (2–10 cm), size 3 (10–25 cm), size 4 (> 25 cm); no size 4 holes observed

**Fig. 2:**
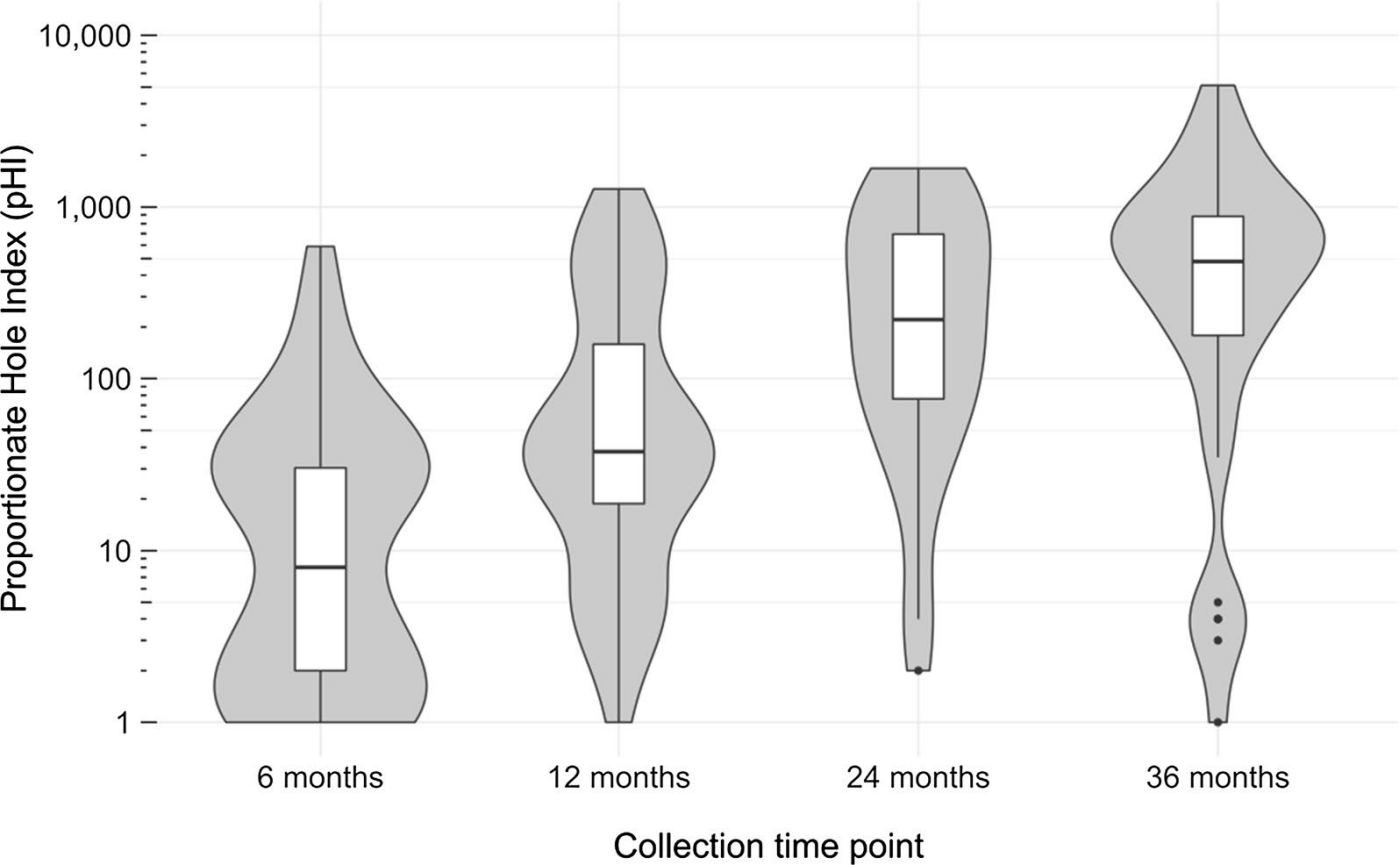
Proportionate hole index (pHI) by collection time point, Waspam, Nicaragua (n = 163 nets). Footnotes: Width of violin-shaped figures represents number of nets with a similar pHI. Boxplots shows the median, interquartile limits, and outliers. The y-axis uses the log_10_ scale

At six months, 78.2 % of nets had at least one hole, increasing to 97.5 % at 12 months, 100 % at 24 months, and 97.1 % at 36 months (Table 5). Only two of the LLINs showed evidence of repairs with stiches along side seams. The proportion of LLINs in the good, damaged, and too torn categories, stratified by collection time point, is presented in Table 5. At six months, 87.3 % of LLINs were physically in good condition and none were too torn. By 36 months, 20.6 % were still in good condition and 38.2 % were too torn. At six months, the median pHI was 7, increasing to 480.5 by 36 months. Significant differences were found when comparing the geometric means of pHI by months of LLIN use, as estimated by the collection time point (ANOVA, p < 0.0001). Post-hoc testing confirmed statistically significant differences between each collection time point (p < 0.05) except when comparing 24 and 36 months (p = 0.86) (Fig. 2).

**Table 5:** Physical condition of LLINs by collection time point, Waspam, Nicaragua (n = 163 nets)

Characteristics	Collection time point			
	**6 months**	**12 months**	**24 months**	**36 months**
LLIN pHI category, n (%)				
Good	48 (87.3)	28 (70.0)	8 (23.5)	7 (20.6)
Damaged	7 (12.7)	9 (22.5)	16 (47.1)	14 (41.2)
Too torn	0 (0.0)	3 (7.5)	10 (29.4)	13 (38.2)
Nets with at least one hole, n (%)	43 (78.2)	39 (97.5)	34 (100.0)	33 (97.1)
Number of holes, median (range)	2.0 (0–26)	6.5 (0–96)	21.0 (1–74)	25.5 (0–160)
pHI, median (range)	7.0 (0–588)	37.5 (0–1,272)	221.0 (1–1,678)	480.5 (0–5,113)
Nets with at least one seam failure, n (%)	10 (18.2)	9 (22.5)	19 (55.9)	11 (32.4)
Repairs, n (%)	0 (0.0)	1 (2.5)	1 (2.9)	0 (0.0)
Total number of nets, n	55	40	34	34
Hole size, n (%)				
Size 1	207 (82.8)	492 (80.0)	671 (77.4)	749 (69.1)
Size 2	37 (14.8)	104 (16.9)	138 (15.9)	232 (21.4)
Size 3	6 (2.4)	19 (3.1)	58 (6.7)	103 (9.5)
Total number of holes, n (%)	250 (100.0)	615 (100.0)	867 (100.0)	1,084 (100.0)

pHI category: good (≤ 64), damaged (65–642), too torn (≥ 643)

Hole size categories: size 1 (0.5–2 cm), size 2 (2–10 cm), size 3 (10–25 cm), size 4 (> 25 cm); no size 4 holes observed

Of the 146 survey respondents that answered the question regarding what caused the most holes in their LLIN, most said holes were caused by children (32.9 %), followed by tucking the net under the mattress (28.1 %), hanging on wire (22.6 %), and animals (10.3 %). Nearly all respondents (98.2 %) reported tucking the net under the bed or mattress at night.

The multivariable pHI model chosen by the LASSO procedure included the age of the LLINs (as estimated by the collection time points), the number of people sleeping in the HH the night before, the water source used to wash nets, the washing method, and whether the net was washed more than six times (p < 0.0001) (Table 6). Because of the small number of LLINs that had evidence of any repairs (n = 2), this variable could not be included for model selection. The interaction term between the age of the LLIN and number of people sleeping in the HH was significant, indicating that the effect of the age of the LLIN on the pHI was dependent on how many people slept in the HH the night before.

**Table 6:** Final linear model^†^ of pHI, Waspam, Nicaragua (n = 163 nets)

Variable	Coefficient (95 % CI)	p-value
Intercept	1.336 (0.673–1.999)	< 0.001*
Collection time point (ref: six months)		
12 months	0.105 (-0.455–0.666)	0.71
24 months	0.513 (-1.139–2.165)	0.54
36 months	1.650 (0.817–2.485)	< 0.001*
No. of people sleeping in the home the night before the survey (ref: 0–4 people)		
5–9 people	− 0.705 (− 1.280– − 0.130)	0.02*
10 + people	− 0.179 (− 0.839–0.480)	0.59
Water source used to wash net (ref: Piped water)		
Well water	0.026 (− 0.296–0.349)	0.87
River water	0.345 (− 0.070–0.760)	0.10
Washed > 6 times (ref: No)		
Yes	0.220 (− 0.081–0.521)	0.15
Washing method (ref: By hand)		
Stone sink/river rock	− 0.203 (− 0.624–0.217)	0.34
Wooden washboard	− 0.149 (− 0.568–0.269)	0.48
Interaction of collection time point and no. of people sleeping in the home (ref: 6 months: 0–4 people)		
12 months: 5–9 people	1.347 (0.267–2.427)	0.01*
24 months: 5–9 people	1.011 (− 0.682–2.704)	0.24
36 months: 5–9 people	− 0.273 (− 1.184–0.637)	0.55
24 months: 10 + people	0.794 (− 1.033–2.662)	0.39
36 months: 10 + people	− 0.496 (− 1.553–0.561)	0.35

* p < 0.05;

^†^ R^2^ = 0.460, F-statistic 8.172, p-value = 3.457 × 10–13

### Cone bioassays

**Fig. 3:**
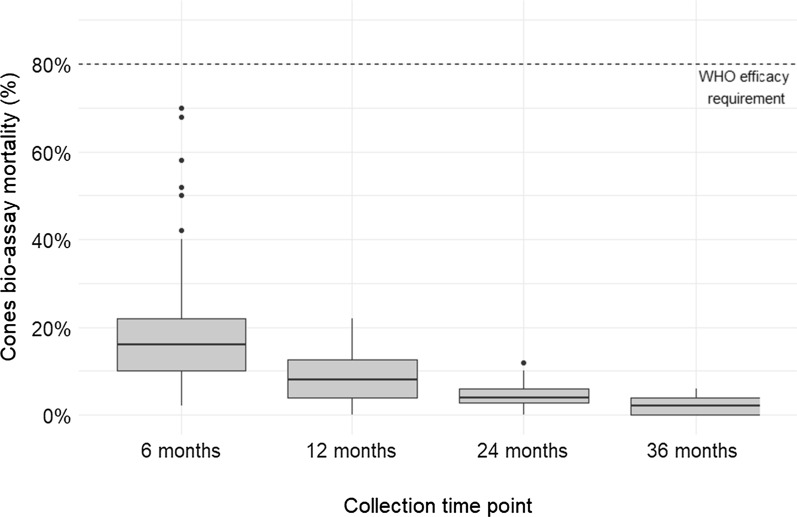
Mosquito mortality by cone bioassay per collection time point, Waspam, Nicaragua (n = 160 nets). Footnote: Estimated mosquito mortality per net calculated by taking the mean of all swatches

After six months of use, only 10 out of 55 tested LLINs had one or more swatches that showed a mortality ≥ 80 % during the cone bioassay. Only 1 out of 40 LLINs tested at 12 months had a swatch with a mortality ≥ 80 %. The mortality of all other swatches of all LLINs tested after 12 months was below the 80 % WHO threshold of optimal efficacy [6]. After six months of use, the median mortality per LLIN was 16 % (range: 2–70 %). By 36 months, the median mortality had decreased to 2 % (range: 0–6 %) (Fig. 3). KD at 60 minutes showed similar results. After six months of use, the median KD per LLIN was 22 % (range: 2–64 %), while by 36 months is has decreased to 0 % (range: 0–6 %).

Significant differences were found when comparing the mean cone bioassay mortality by months of LLIN use, as estimated by collection time point (p < 0.0001). Further pair-wise comparisons confirmed a statistically significant difference between each collection time point (p < 0.05) except when comparing 12 and 24 months (p = 0.26), and 24 and 36 months (p = 0.59).

### Insecticide content

Due to equipment restrictions, only 26 out of the 55 LLINs collected at 6-months post distribution were analyzed by HPLC for insecticide content. The mean total deltamethrin content after 6 months of use was 31.9 mg/m^2^ (range: 10.0–55.3 mg/m^2^). None of the LLINs tested were under the minimum effective concentration of 4 mg/m^2^, and only three were lower than the suggested optimum concentration of 15 mg/m^2^ [16]. Over half (73.1 %) of the nets were reported to have been washed more than once per month, and those maintained a mean deltamethrin concentration of 30.3 mg/m^2^. LLINs that were washed once per month (n = 4) had a mean deltamethrin concentration of 32.3 mg/m^2^, and the single LLIN that had not been washed had a deltamethrin concentration of 33.4 mg/m^2^.

**Fig. 4:**
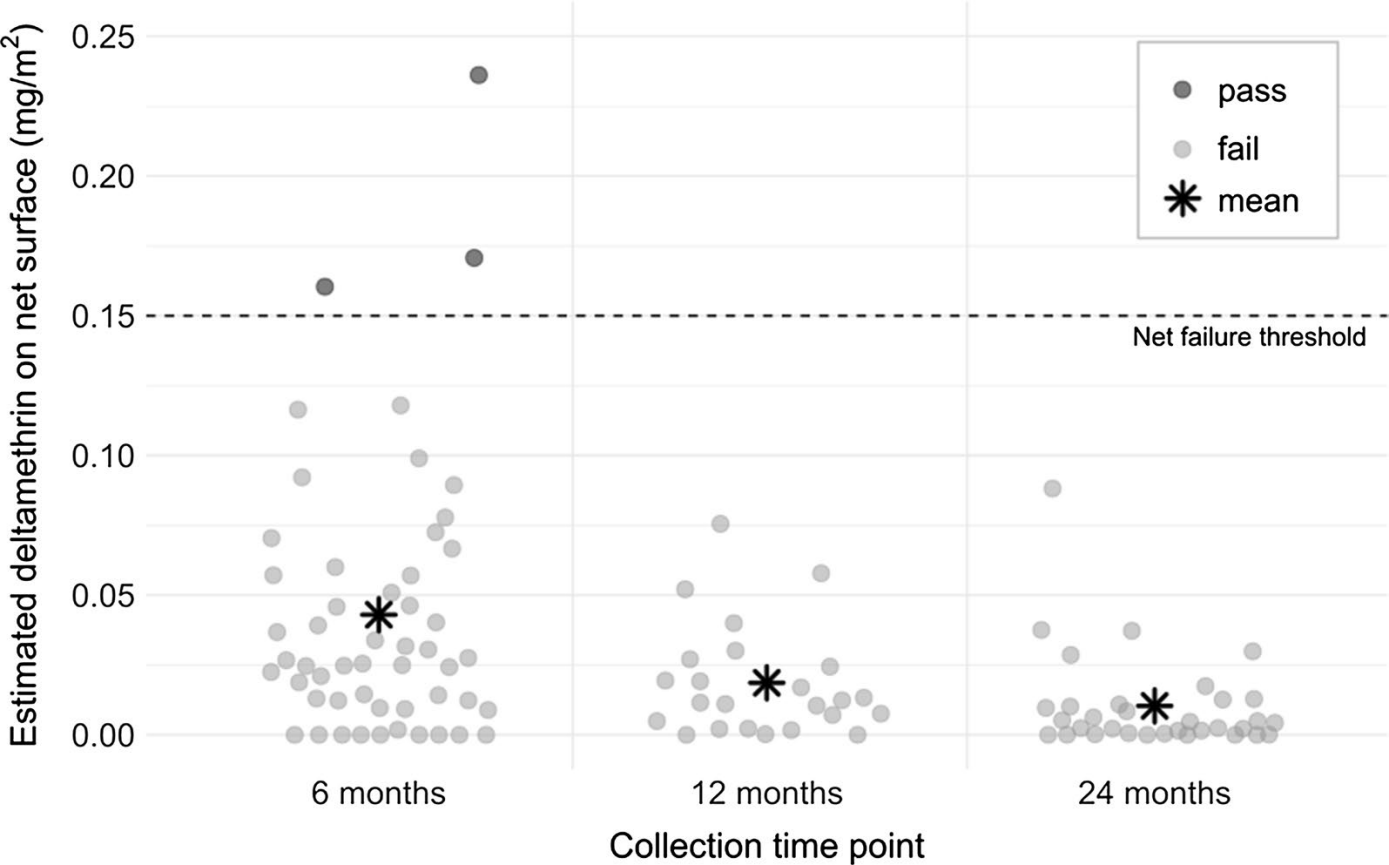
Cyanopyrethroid field test results per collection time point, Waspam, Nicaragua (n = 109 nets). Footnote: Threshold for net failure set at 0.15 mg/m^2^, equivalent to the surface concentration required to achieve 80 % mortality with susceptible *Anopheles gambiae* in cone bioassays

CFT analysis was conducted on 52 LLINs collected at six months, 24 nets at 12 months and 33 nets at 24 months. There was a rapid decrease in the level of deltamethrin detected on the surface of the LLINs. After 6 months of use, 94.2 % of the LLINs had CFT results that crossed the threshold for being considered a failed net (0.15 mg/m^2^), while 100 % failed at 12 and 24 months (Fig. 4).

**Fig. 5:**
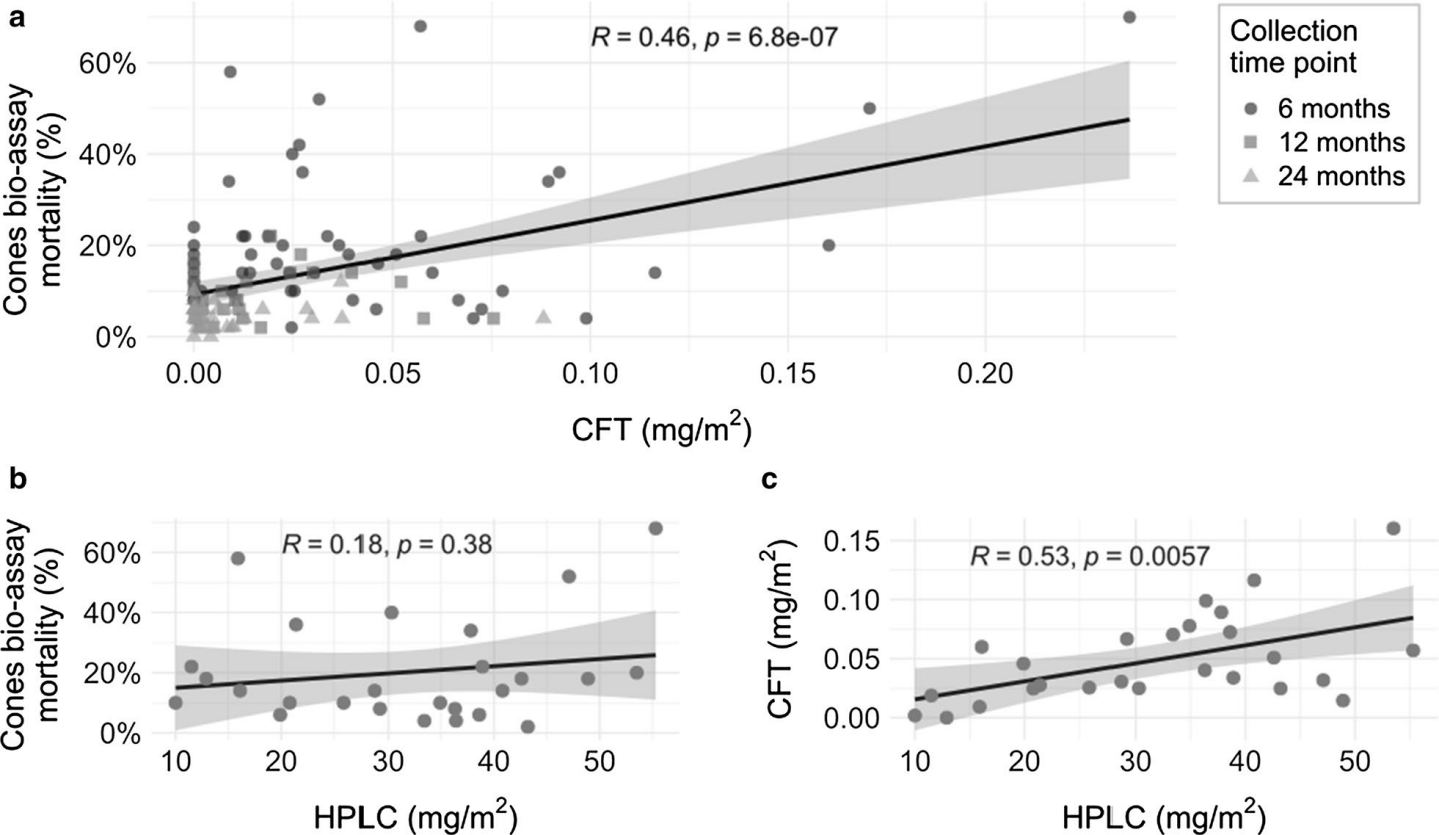
Pairwise-comparisons between high-performance liquid chromatography (HPLC), cyanopyrethroid field test (CFT), and cone bioassay results from LLINs, Waspam, Nicaragua (a, n = 108 nets; b, n = 26 nets; c, n = 26 nets). Footnote: HPLC comparison considered only LLINs collected at 6 months

Figure 5 summarizes the pairwise-comparisons between results obtained from CFT, HPLC, and cone bioassays. When the results from CFT and HPLC were compared, a statistically significant positive correlation was detected (r = 0.53, p < 0.01). When CFT results were compared with results from the cone bioassays, a significant positive correlation was detected (r = 0.46, p < 0.001). However, no correlation was detected between HPLC and cone bioassay results (r = 0.18, p = 0.38).

## Discussion

As observed in similar studies conducted in Africa, the physical conditions of the LLINs distributed in Nicaragua deteriorated over time. The most notable drop in LLIN physical integrity happened between the 12-month and the 24-month sampling periods, although most nets had evidence of holes even after only six months of use. This loss of physical integrity was also reflected in a significant increase in the pHI between those two time points; at 24-months, 76.5 % LLINs were classified as damaged or too torn, compared to only 30 % at 12 months. This is comparable to observations reported by a similar LLIN study in Ethiopia, and highlights the limited physical lifetime of LLINs [17].

The reported handling of the LLINs was associated with reduced physical durability. From the HH interviews, the holes in nets were most commonly attributed to mishandling by children, tucking them under mattresses, or rips due to being dried on wire. Similar to what has been reported elsewhere, the highest concentration of holes was detected near the bottom of the LLINs [17]. Tucking nets under mattresses likely contributes to holes forming on the lower third of the net, but is a critical practice for preventing mosquitoes from entering the nets [3, 18–20]. It is important for both manufacturers and end-users to know where physical damage is most likely to occur, as this could allow for nets to be reinforced in such areas, and for users to be better guided in their handling and repair efforts. Prior laboratory studies have shown that *Anopheles albimanus* more often attack the roof of nets, which means that even small holes in the roof panel might pose a greater risk than damage on the lateral panels [21]. To what extent this laboratory mosquito behaviour is comparable to wild *An. albimanus* in Central America is yet to be determined.

There was little evidence of repairs made to damaged LLINs in this study, in contrast to what has been reported from similar studies in Africa [2, 22]. It was also interesting to note that the perceived mosquito protection benefit was highest at 36 months, when LLINs were already physically damaged, rather than soon after distribution. These observations underscore the need for strong messaging on net care and promotion of routine use at time of distribution. Unfortunately, this study did not evaluate LLIN attrition over the months, which is a major limitation to interpret net durability data. However, we found a campaign net in 98.8 % of HHs where interviews were conducted, possibly signaling a high net retention rate over the months.

This study detected a progressive increase in pHI at each evaluation time-point. However, this difference was not statistically significant between 24 and 36 months, suggesting that the physical damage to the nets had peaked at 24 months. The extent to which the physical damage to the LLINs studied in Nicaragua affected their ability to protect against malaria transmission was not quantified in this study. A previous study in Malawi showed that after one year of use, nets were still classified as being in good condition by WHO criteria and there was no association between malaria and the total area of holes on the nets [23]. If those observations hold true for the epidemiological conditions in the Americas, one may assume that, since the majority of LLINs in this study were also in good condition up to 12 months, that they remained adequately able to protect the population at risk. However, given their subsequent physical deterioration and loss of chemical efficacy, it is unclear the extent to which those factors may have resulted in an increased risk of malaria after 12 months of use.

The frequency of LLIN washing observed in this study and the fact that many LLINs were exposed to sunlight while drying could have led to the unexpectedly low bioefficacy detected through the cone bioassays at 6-month post distribution, which has been demonstrated in other studies [4, 5, 18, 20, 24, 25]. Despite the low mortality in the bioassays at six months, the HPLC results suggested that sufficient insecticide remained on the nets for them to be considered functioning at an optimal level. However, HPLC analysis extracts the total insecticide content from a piece of net, so it is unclear what proportion of that insecticide is actually bioavailable to mosquitoes alighting on the net surface. Results from the CFT showed that nearly all of the nets analysed at 6 months would be considered ‘failed’ due to extremely low surface levels of deltamethrin. According to the manufacturer, the total amount of deltamethrin in a new, unused PermaNet 2.0^®^ is 55 mg/m^2^ ± 25 %. Previous research using the CFT to detect deltamethrin on the surface of new PermaNet 2.0^®^ resulted in 1.01 mg/m^2^ (95 % CI:0.94–1.09) and associated a value of 0.15 mg/m^2^ with a mortality of 80 % in the cone bioassay. A net containing less than 15 % (0.15/1.01) deltamethrin relative to a new net was designated as the threshold value representing a failed net [24].

The bioassay results were surprising, as they showed very low mortality rates even after only 6 months of use. Permanet 2.0® have previously been shown to retain insecticidal efficacy after 20 washes and 2–3 years of use [25–27]. It is worrisome that LLINs with only 6 months of use had such low mortality rates. It is not clear if this finding can be explained by nets that arrived for use already with some degree of insecticide deterioration, as LLINs were not evaluated prior to delivery to the HHs. In addition to stringent quality control and assurance programs during net manufacturing and pre-delivery, these findings highlight the need for methods that are field practical and would allow for simple and robust routine analyses of nets from the time of distribution through their expected lifespans to quickly detect deterioration [10].

The results presented here provide the first comprehensive analysis of LLIN durability in a malaria elimination setting in Central America. This study shows that over 36 months of follow-up, LLINs quickly lost chemical bioefficacy and progressively became more physically damaged. These results can be used to guide future LLIN interventions in malaria elimination settings in Nicaragua and potentially elsewhere in Central America, and highlight the importance of educating the populations that receive the LLINs on best practices regarding their care and maintenance. An important limitation of this study is the lack of entomological data to substantiate the extent to which LLINs were able to protect the target population from bites of local malaria vectors. *Anopheles* species present in Central America are known to exhibit heterogeneous biting behaviours, often with peak biting times that occur outside of the hours when people are most likely to be protected under nets [28–31]. As vector control continues to be focused on malaria elimination settings in the Americas, achieving a well-rounded understanding of the factors that influence the efficacy of different vector control tools in these settings will be key to their successful implementation in achieving their maximum impact.

## Data Availability

The datasets used and/or analysed during the current study are not provided.
